# Sodium glucose co-transporter 2 inhibitors in the treatment of glomerular diseases: a *CKJ* controversy

**DOI:** 10.1093/ckj/sfae237

**Published:** 2024-08-12

**Authors:** Fernando Caravaca-Fontán, Lucia del Vecchio, Manuel Praga, Jürgen Floege, Carmine Zoccali

**Affiliations:** Department of Nephrology, Instituto de Investigación Hospital 12 de Octubre (imas12), Madrid, Spain; Department of Nephrology and Dialysis, ASST Lariana, Como, Italy; Department of Medicine, Complutense University, Madrid, Spain; Department of Nephrology and Rheumatology and Department of Cardiology, RWTH University Hospital Aachen, Aachen, Germany; Renal Research Institute, NY, USA; Institute of Molecular Biology and Genetics, (Biogem), Ariano Irpino, Italy; Associazione Ipertensione Nefrologia Trapianto Renale (IPNET), c/o Nefrologia, Grande Ospedale Metropolitano, Reggio Calabria, Italy

**Keywords:** chronic kidney disease, eGFR, progression, proteinuria, SGLT2i

## Abstract

Integrating sodium-glucose co-transporter 2 inhibitors (SGLT2i) into the treatment for chronic kidney disease (CKD) has marked a significant therapeutic advance in nephrology. Clinical trials such as DAPA-CKD and EMPA-KIDNEY have demonstrated the beneficial effects of SGLT2i in slowing CKD progression and reducing proteinuria. However, the applicability of these results to patients with glomerulonephritis is still unresolved due to various limitations. This manuscript combines the evidence supporting the use of SGLT2i in glomerular diseases, highlights the limitations and strikes a conclusive balance on their role in clinical practice.

## INTRODUCTION

In recent years, the integration of sodium-glucose co-transporter 2 inhibitors (SGLT2i) into the treatment landscape for chronic kidney disease (CKD) and glomerular diseases has marked a significant advance in nephrology. Large randomized controlled clinical trials such as DAPA-CKD [1, 2] and EMPA-KIDNEY [3] have consistently demonstrated the beneficial effects of SGLT2i in slowing CKD progression and reducing proteinuria beyond diabetic nephropathy. However, the applicability of these results to patients with glomerular diseases is suboptimal for several reasons. This manuscript aims to combine the evidence supporting the use of SGLT2i in glomerular diseases, highlights the strengths and weaknesses of the current data, and strikes a conclusive balance on their role in clinical practice.

## PRO PERSPECTIVE

Drs Fernando Caravaca-Fontán and Manuel Praga argue that SGLT2i bring a major benefit in the treatment of glomerular diseases. They remark that large-scale trials such as DAPA-CKD and EMPA-KIDNEY have shown that SGLT2i reduce the risk of CKD progression and cardiovascular death in patients with CKD, including those with glomerular diseases. Specific sub-analyses of these trials in patients with immunoglobulin A nephropathy (IgAN) or focal segmental glomerulosclerosis (FSGS) have demonstrated benefits in terms of reductions in proteinuria and/or estimated glomerular filtration rate (eGFR) decline. Observational studies have shown that SGLT2i can significantly reduce proteinuria and slow eGFR decline in patients with various glomerular diseases, even those with persistent proteinuria, despite other treatments [4]. SGLT2i have been associated with additional therapeutic benefits, such as reducing the risk of hyperkalemia [5], modestly lowering blood pressure and offering weight loss benefits.

The DAPA-CKD trial included 270 participants with IgAN, 254 (94%) of whom had biopsy-proven diagnosis. The mean eGFR was 43.8 mL/min/1.73 m^2^, and the median urinary albumin-to-creatinine ratio (ACR) was 900 mg/g. Patients were followed for a median of 2.1 years. The primary composite outcome was less common in the dapagliflozin arm compared with placebo [4% vs 20%, hazard ratio (HR) 0.29, 95% confidence interval (CI) 0.12–0.73], and patients assigned to dapagliflozin had a significantly slower mean annualized eGFR decline (–3.5 vs –4.7 mL/min/1.73 m^2^). Moreover, dapagliflozin resulted in a 26% reduction in albuminuria compared with placebo, with a favourable safety profile. Thus, in this sub-analysis, the addition of dapagliflozin alongside stable renin–angiotensin system (RAS) inhibitors substantially attenuated the risk of CKD progression [1].

Another sub-analysis of the same trial comprising patients FSGS [2] evaluated the efficacy and safety of dapagliflozin in patients with biopsy-proven diagnosis. Of 104 patients, 45 (43%) were randomized to dapagliflozin and 59 (57%) to placebo. The mean eGFR was 41.9 mL/min/1.73 m^2^ and the median ACR was 1248 mg/g [interquartile range (IQR) 749–2211]. Although the primary composite outcome did not reach statistical significance (HR 0.62, 95% CI 0.17–2.17), participants treated with dapagliflozin had a 26.1% reduction in albuminuria compared with 9.9% with placebo, which persisted after a year. In addition, the mean annual rate of chronic eGFR decline was lower in participants who received dapagliflozin (–1.9 mL/min/1.73 m^2^, 95% CI –3.0 to –0.9) compared with placebo (–4.0 mL/min/1.73 m^2^, 95% CI –4.9 to –3.0) [2].

The EMPA-KIDNEY trial [3] evaluated the effects of empagliflozin in 6609 participants with CKD, 1669 (25.3%) of whom had investigator-reported glomerular disease: 817 with IgAN and IgA vasculitis, 195 with primary and secondary FSGS, 96 membranous nephropathy, 27 lupus nephritis, 17 granulomatosis with polyangiitis, 14 microscopic polyangiitis and 13 with membranoproliferative glomerulonephritis, among other diseases. The mean ± standard deviation eGFR among patients with glomerular disease was 42.4 ± 17.8 mL/min/1.73 m^2^, and the median ACR was 700 mg/g (IQR 306–1428). The primary composite outcome of kidney disease progression or cardiovascular death occurred in 117 (13.7%) patients with glomerular diseases in the empagliflozin group and 558 (16.9%) in the placebo group (HR 0.77, 95% CI 0.60–0.98), with broadly similar effects across the other major CKD disease aetiologies. The relative reductions in the chronic rate of eGFR decline were notable across different glomerular disease groups: –43% (95% CI –59 to –27) in participants with IgAN, –22% (–60 to 16) in individuals with FSGS and –41% (–62 to –21) in patients with other causes of glomerular disease. Furthermore, the proportional reduction in study average ACR compared with placebo was –24% (IQR –33 to –13%), –23% (IQR –42% to 2%) and –2% (IQR –17 to 15%), respectively, in the three disease groups. Hence, in this trial, empagliflozin treatment was associated with reduced risk of kidney disease progression and eGFR decline, exhibiting comparable relative effects regardless of the underlying primary kidney disease aetiology. These findings, along with those from another prespecified analysis of the same trial showing that empagliflozin reduced the rate of eGFR decline even in patients with minimal albuminuria, provide robust evidence of the protective effects of SGLT2i in a broad spectrum of patients with CKD, including those with glomerular diseases [3].

**Figure 1:**
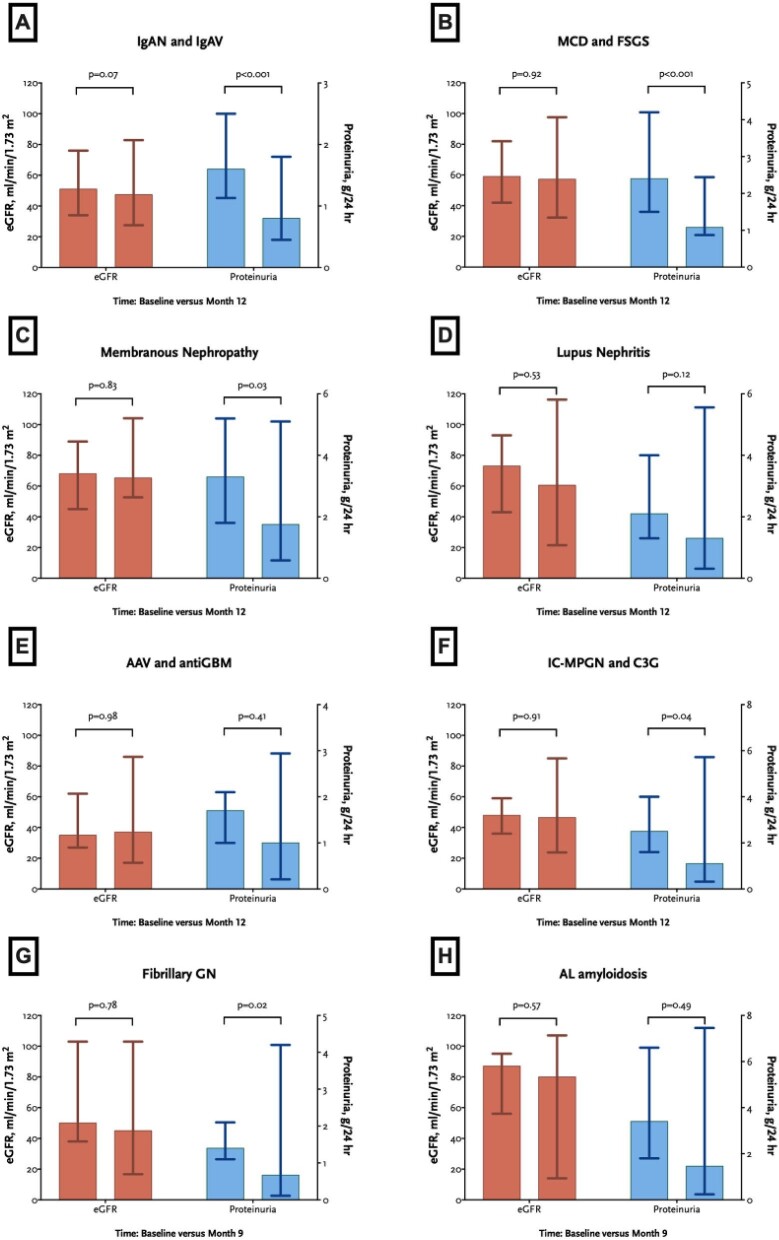
Boxplots with the absolute difference between baseline and 9- or 12-month eGFR and proteinuria, according to underlying etiologies, in the real-world clinical study by Caravaca-Fontán *et al*. [4].

The growing evidence for the nephroprotective effects of SGLT2i beyond diabetic nephropathy has catalysed their integration into clinical practice in the last few years, particularly as an adjunctive therapy for patients with glomerular diseases and persistent proteinuria despite renin–angiotensin therapy or diuretics. This ‘real world’ clinical experience with the use of SGLT2i in glomerular diseases was collected in an international, multicentric, observational cohort study coordinated by the Immunonephrology Working Group of the European Renal Association (ERA) [4] (Fig. 1). Four-hundred and ninety-three adult patients with biopsy-proven glomerular diseases, persistent residual proteinuria ≥1 g/day despite targeted immunosuppression and background therapy with RAS inhibitors were enrolled. These included 203 (42%) patients with IgAN and IgA vasculitis, 90 (18%) primary and secondary FSGS, 89 (18%) with membranous nephropathy and 32 (7%) with lupus nephritis, among others. The geometric mean percentage change of proteinuria decreased by –35%, –41%, –45% and –48% at 3, 6, 9 and 12 months following the initiation of SGLT2i, while eGFR declined by –6%, –3%, –8% and –10.5% at corresponding intervals. Interestingly, these changes were consistent across all the various underlying diseases, suggesting a potential beneficial cross-sectional effect within this spectrum of conditions [4].

Participants with a serum albumin <3.5 g/dL at SGLT2i initiation were less likely to achieve a ≥30% reduction in proteinuria during follow-up, indicating limited response in those with active nephrotic syndrome. Based on that, it can be hypothesized that serum albumin may serve as a discriminative factor, distinguishing between patients exhibiting residual proteinuria primarily due to hyperfiltration (who are likely to show improved antiproteinuric response to RAS blockade or SGLT2i) and those with more pronounced impairment of glomerular permselectivity resulting from other pathogenic mechanisms (who may exhibit a less robust antiproteinuric response to RAS blockade or SGLT2i).

On the other hand, patients with higher body mass index exhibited a more substantial percentage reduction in proteinuria. Obesity is a well-recognized predictor of kidney disease progression, with persistent glomerular hyperfiltration widely accepted as one of the primary pathogenic mechanisms driving obesity-related nephropathies. These findings strongly suggest the pivotal role of hyperfiltration abrogation as a central mechanism for reducing proteinuria with these drugs.

Finally, consistent with the results in clinical trials, patients who achieved a ≥30% proteinuria reduction after SGLT2i initiation had a slower eGFR decline over time. Hence, this clinical experience demonstrated that adding SGLT2i to conventional antiproteinuric therapy improved proteinuria, regardless of the underlying aetiology. Despite this significant reduction, 271 patients (55%) still exhibited proteinuria >1 g/day at their last follow-up. This highlights an unmet need for optimal antiproteinuric response in certain patient profiles and suggests the potential for newer, synergistic therapeutic approaches.

Numerous additional therapeutic benefits have been associated with SGLT2i, offering potential therapeutic value for patients with glomerular disease. RAS blockade is a cornerstone in treating glomerular diseases, effectively controlling blood pressure and proteinuria while offering other cardiovascular benefits. However, RAS blockade can promote the development of hyperkalemia, especially when combined with mineralocorticoid receptor antagonists (MRAs), potentially limiting their use or the achievable target doses in patients with glomerular diseases. In this context, SGLT2i may offer an opportunity to enhance potassium excretion, given their primary tubular site of action. This was evaluated in a meta-analysis involving six SGLT2i clinical trials in patients with type 2 diabetes and high cardiovascular risk or CKD. SGLT2i consistently reduced the risk of serious hyperkalemia—defined as serum potassium >6 mmol/L—across a range of subgroups, including baseline kidney function and history of heart failure, and use of RAS blockade, diuretics and MRAs [5]. Another randomized crossover trial comprising 46 patients with CKD assessed the efficacy and safety of dapagliflozin and eplerenone alone or combined. After 4 weeks of treatment with dapagliflozin alone, eplerenone alone or the combination of dapagliflozin–eplerenone there was –19.6%, –33.7% and –53% mean percentage change of ACR from baseline. The incidence of hyperkalemia was lower with the combination therapy compared with eplerenone alone [6]. Thus, based on this evidence, it now appears legitimate to recommend using SGLT2i to reduce the risk of hyperkalemia induced by antiproteinuric and kidney protective agents.

SGLT2i modestly lower both systolic and diastolic blood pressure without significantly increasing the risk of hypotensive episodes, and they also offer modest benefits for weight loss. On the other hand, recent insights from animal models have revealed the potential nephroprotective effects of SGLT2i in lupus mice. These effects include alleviating podocyte injury by reducing inflammation and enhancing autophagy through reduction of mammalian target of rapamycin complex 1 (mTORC1) pathway activity, which plays a crucial role in the pathogenesis of regulatory T-cell dysfunction in systemic lupus erythematosus [7]. Furthermore, animal models have also suggested the potential effects of SGLT2i on the complement system, although results have not been entirely consistent [8].

## CON PERSPECTIVE

**Figure 2:**
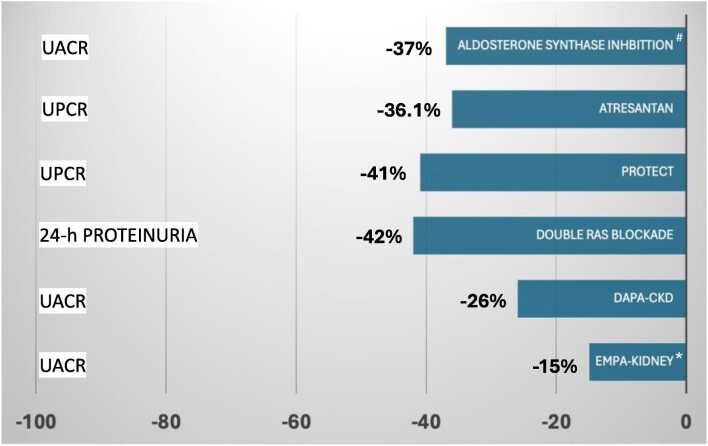
Percentage reduction in difference in urinary protein to creatinine ratio or ACR with SGLT2i and other nephroprotective agents compared with placebo in IgA nephropathy or non-diabetic chronic kidney disease. UACR: urine ACR; UPCR: urine protein to creatinine ratio. Data for atresantan were obtained from the interim analysis of the ALIGN trial (NCT04573478), a phase III study of 270 patients with IgAN (https://www.novartis.com/news/media-releases/novartis-atrasentan-phase-iii-data-show-clinically-meaningful-proteinuria-reduction-further-advancing-companys-iga-nephropathy-igan-portfolio). Data for sparsentan were obtained from the PROTECT study (NCT03762850) of 406 patients with IgAN [11]. Data for double RAS blockade come from a metanalysis of six studies in patients with IgAN (*n* = 106) [12]. The aldosterone synthase inhibitor BI 690517 was tested in comparison with placebo in a phase 2 trial (NCT05182840) of 586 patients with non-diabetic CKD either receiving or not empagliflozin. The antiproteinuric effect refers to patient not treated with empagliflozin.

Dr Lucia del Vecchio cautions that the evidence for the use of SGLT2i in glomerular diseases is limited. She highlights that the secondary analyses of glomerular disease in these trials showed wide CIs for some primary composite outcomes with no significant reduction in the risk of kidney failure in EMPA-KIDNEY, probably because they were underpowered following anticipated closure. The antiproteinuric effect of SGLT2i is mild compared with other nephroprotective agents (Fig. 2), and a significant proportion of patients showed no effect. The safety of SGLT2i during active immunosuppression is still to be verified, and there are concerns about the risk of infections and other adverse events.

The applicability of DAPA-CKD and EMPA-KIDNEY results to patients with glomerulonephritis is suboptimal for several reasons. Patients with normal renal function were excluded; those with proteinuria in the nephrotic range were represented little. The same holds true for patients of Black or African-American race and those on active immunosuppression. In addition, while the two trials included many patients with IgA nephropathy and focal segmental glomerulonephritis, other types of glomerulonephritis were under-represented. Finally, the antiproteinuric effect of SGLT2i is mild compared with other nephroprotective agents.

There is strong evidence that SGLT2i effectively provide renal and cardiovascular protection in patients with non-diabetic kidney disease. Despite this premise, patients with glomerular diseases differ from the overall population in which SGLT2i have been tested, leaving some gaps in knowledge that still need to be filled.

The secondary analyses of glomerular disease in EMPA-KIDNEY showed that the benefit of the primary composite outcome or any progression of kidney disease was of borderline significance. The risk of kidney failure was not even significant. These weak results can be explained by the fact that the trial was underpowered for secondary analyses, also considering that it was stopped prematurely. This weakness is further highlighted when calculating the number needed to treat (NNT) and the absolute risk reduction: only 28 for the primary composite endpoint, but with a wide 95% CI (16–647).

The data for glomerular disease are stronger in DAPA-CKD (HR 0.43, 95% CI 0.26–0.71 for the primary outcome), probably because it enrolled a higher percentage of patients with more advanced and thus more progressive CKD.

The lack of statistical power is even more evident when looking at individual cardiovascular events, as only 13 patients with glomerulonephritis in EMPA-KIDNEY were hospitalized for heart failure or died from cardiovascular causes [3]. This is due to the low burden of cardiovascular disease in this relatively young patient population, the majority of whom do not have diabetes, heart failure or peripheral vascular disease at enrolment. Similar data were found in DAPA-CKD: only 24 patients had a history of heart failure, and only 34 (0.24%) were hospitalized for heart failure or died of cardiovascular causes during follow-up [1].

In recent years, the use of the slope of serum creatinine over time has been validated as a surrogate endpoint for clinical trials in patients with glomerulonephritis to increase the likelihood of demonstrating the effect of a given drug, particularly in the early stages of rare diseases such as glomerulonephritis. The chronic slope is the choice when there is an initial acute drop in eGFR, as with SGLT2i. In this respect, both trials showed a significant favourable effect in reducing the eGFR decline in patients with primary glomerulonephritis.

Along with the slope of eGFR, the percentage reduction in proteinuria has also been validated as a surrogate endpoint, with a 30% reduction considered the gold standard for efficacy, especially in patients with high baseline albuminuria. This appears to be the case also for SGLT2i, as an exploratory analysis showed that albuminuria reduction was the most important measured determinant of the benefit observed in EMPA-KIDNEY, explaining one-fifth of the effect on the chronic slope [3].

In patients with glomerular disease, the difference in the antiproteinuric effect of SGLT2i compared with placebo is small. In EMPA-KIDNEY, the reduction was –15% (95% CI –24% to –6%), and in DAPA-CKD, it was –13.6% (95% CI –24.9% to –0.6%). The CIs are wide and close to zero, indicating that a significant proportion of patients had no effect. Furthermore, lower systolic blood pressure levels during follow-up may have contributed to the antiproteinuric effect (–2.3 mmHg, 95% CI 1.2–3.4 mmHg, in patients without diabetes receiving dapagliflozin).

### Evidence in specific glomerular diseases

DAPA-CKD and EMPA-KIDNEY enrolled a variety of primary and secondary glomerulonephritis, with a significant proportion not biopsy-proven (21.4% in EMPA-KIDNEY and at least 7% in DAPA-CKD). This increases the uncertainty of the results obtained when dealing with specific forms of glomerulonephritis.

Patients with IgAN represent the largest category of patients with glomerular diseases in the DAPA-CKD and EMPA-KIDNEY trials (270 in the first [1, 2] and 817 in the second [3]). A pre-specified secondary analysis of DAPA-CKD confirmed the efficacy of dapagliflozin in this cohort, but with some limitations [1]. First, the number of patients reaching the primary outcome was rather low [only 6 (4%) and 20 (15%) in the dapagliflozin and placebo groups, respectively]. The observed benefit is largely offset by the higher number of patients with an event in the placebo group. It is then possible that suboptimal supportive care was offered [9]. This is of importance, considering that maximal RAS inhibition, optimal blood pressure control, dietary sodium restriction and healthy lifestyle may have a substantial effect on IgAN stabilization, similar to that obtained with immunosuppression [10].

In addition, the incidence of the primary composite outcome was 3.5-fold higher in patients with an eGFR <45 mL/min/1.73 m^2^, implying a lack of events and therefore conclusiveness in those with a more preserved eGFR. Similar considerations apply to urinary ACR levels and the other key secondary endpoints.

Available data from EMPA-KIDNEY specific to IgAN showed a significant effect on the chronic slope compared with placebo (absolute difference –1.78 mL/min/1.73 m^2^, 95% CI 1.13–2.44), possibly due to the larger sample size [3]. Notably, also in this trial, requirements for maximal RAS inhibition before randomization were suboptimal (the protocol required only a clinically appropriate dose without mentioning stability).

The second most common type of glomerulonephritis is FSGS (*n* = 104 in DAPA-CKD and *n* = 317 in EMPA-KIDNEY). The study of drug efficacy in FSGS is complicated by its heterogeneity and the difficulty of distinguishing primary from secondary forms. In this respect, EMPA-KIDNEY separated the two categories. However, it is not known whether all cases were biopsy-proven and whether all patients underwent electron microscopy. Both dapagliflozin and empagliflozin showed a trend towards a lower rate of eGFR decline as a chronic slope, without reaching statistical significance. Moreover, in DAPA-CKD, the antiproteinuric effect was mild [between-group difference of 19.7% (standard error 9.0) for dapagliflozin versus placebo].

Glomerulonephritis other than IgAN or FSGS are much less represented in the two trials, and some were not even included, such as lupus nephritis and vasculitis in DAPA-CKD. As a result, the degree of evidence for these individual glomerulonephritis remains scanty despite the assumption that SGLT2i are generally effective.

## THE MODERATORS’ PERSPECTIVE

To strike a balance, the *CKJ* Editor-in-Chief, Dr Juergen Floege, and a Senior Editor, Dr Carmine Zoccali, will focus on two points to then give their conclusions and perspective.

- Are SGLT2i more effective than other specific treatments for glomerulonephritis?

This question will likely remain unanswered until sufficient data for adequately powered network meta-analyses become available. SGLT2i are now considered the standard of care and will be included in all future trials as supportive therapy and RAS blockers.

Currently, IgAN is the glomerulonephritis with the most active clinical research, with several treatments in clinical development or recently approved. The antiproteinuric effect of SGLT2i appears to be lower than that of Nefecon, endothelin A receptor blockers, complement inhibitors or lymphocyte modulators [11]. In addition, there are concerns that RAS blockade may have been underused or not fully optimized in some of the SLGT2i trials [9]. Nevertheless, even if SGLT2i only exert a modest antiproteinuric effect in patients with glomerulonephritis, they seem to be particularly safe in this population [9].

- Can we use SGLT2i during active immunosuppression?

DAPA-CKD and EMPA-KIDNEY excluded patients with recent immunosuppressive therapy. Only small studies have been published combining SGLT2i with immunosuppressants [13, 14]; they showed a low rate of urinary tract infections successfully treated with antibiotics but a relatively high rate of drug discontinuation due to adverse events. More information will be available from ongoing trials, in particular in IgAN, where SGLT2i are often included as standard of care or where dedicated studies testing combinations are under way. At present caution should be exercised older patients, in particular those with a history of recurrent urinary tract infections.

SGLT2i affect the tubular transport of calcium, magnesium, phosphate and bicarbonate, and it has been suggested that their use may lead to an increased risk of fracture. Although so far this has not been confirmed by meta-analyses, more data are needed in patients who have received or are being actively treated with steroids in addition to SGLT2i.

## CONCLUSIONS AND PERSPECTIVE

Overall, the evidence on the benefits of SGLT2i progressively decreases at higher eGFR, with no data for improved kidney outcomes in patients with preserved kidney function (theoretically those in whom we would like more to prevent progression) and those with proteinuria in the nephrotic range. The antiproteinuric effect of SGLT2i is mild in many instances, possibly because the disease is often sustained by immunological factors that are not affected by SGLT2i. The safety of SGLT2i during active immunosuppression is still to be verified. Nevertheless, on balance, so far the safety of SGLT2i in patients with primary glomerulonephritis is high and they constitute a more than welcome addition to the management of such patients.
